# Endovascular correction of isolated descending thoracic aortic disease: a descriptive analysis of 1,344 procedures over 10 years in the public health system of São Paulo

**DOI:** 10.6061/clinics/2021/e2332

**Published:** 2021-02-01

**Authors:** Maria Fernanda Cassino Portugal, Marcelo Passos Teivelis, Marcelo Fiorelli Alexandrino da Silva, Nickolas Stabellini, Alexandre Fioranelli, Claudia Szlejf, Edson Amaro, Nelson Wolosker

**Affiliations:** I Hospital Israelita Albert Einstein, Sao Paulo, SP, BR; II Faculdade Israelita de Ciencias da Saude Albert Einstein, Sao Paulo, SP, BR; III Faculdade de Ciencias Medicas, Santa Casa de Sao Paulo, Sao Paulo, SP, BR; IV Faculdade de Medicina FMUSP, Universidade de Sao Paulo, Sao Paulo, SP, BR

**Keywords:** Aortic Aneurysm, Aneurysm, Surgery, Aorta, Thoracic

## Abstract

**OBJECTIVES:**

In Brazil, descending thoracic aorta disease (TAD), including aneurysms and dissection, are preferentially managed by endovascular treatment (TEVAR) due to the feasibility and good results of this technique. In this study, we analyzed endovascular treatment of isolated TAD (ITAD) in the public health system over a 10-year period in São Paulo, a municipality in Brazil in which more than 5 million inhabitants depend on the governmental health system.

**METHODS:**

Public data from procedures performed between 2008 and 2019 were extracted using web scraping techniques. The following types of data were analyzed: demographic data, operative technique, elective or urgent status, number of surgeries, in-hospital mortality, length of hospital stay, mean length of stay in the intensive care unit, and reimbursement values paid by the government. Trauma cases and congenital diseases were excluded.

**RESULTS:**

A total of 1,344 procedures were analyzed; most patients were male and aged ≥65 years. Most individuals had a residential address registered in the city. Approximately one-third of all surgeries were urgent cases. There were 128 in-hospital deaths (9.52%), and in-hospital mortality was lower for elective than for urgent surgeries (7.29% *vs.* 14.31%, *p*=0.031). A total of R$ 24.766.008,61 was paid; an average of R$ 17.222,98 per elective procedure and R$ 18.558,68 per urgent procedure. Urgent procedures were significantly more expensive than elective surgeries (*p*=0.029).

**CONCLUSION:**

Over a 10-year period, the total cost of ITAD interventions was R$ 24.766.008,61, which was paid from the governmental system. Elective procedures were associated with lower mortality and lower investment from the health system when compared to those performed in an urgent scenario.

## INTRODUCTION

Thoracic aortic disease (TAD) encompasses aneurysms and dissections. An aneurysm is defined as a localized and permanent dilatation of an artery by at least 50% of its normal diameter (1-3). Dissections are splitting processes of the aortic media that may evolve to dilatations, which may also require corrective surgery to avoid rupture (4). For descending thoracic aorta, current guidelines recommend open repair for patients with chronic dissection and a descending thoracic aortic diameter exceeding 5.5 cm, particularly if associated with a connective tissue disorder, but without significant comorbid disease (5).

Aneurysms of the thoracic aorta may develop in any combination of its four segments: the aortic root, the ascending aorta, the aortic arch, and the descending aorta.

The estimated incidence of TAD is six cases per 100,000 person-years (6). In Brazil, a retrospective analysis found that the prevalence of thoracic aorta aneurysms (TAAs) was 1.08% (7).

Currently, therapeutic alternatives for descending TAD include open surgical repair, and endovascular treatment (TEVAR) for large aneurysms (>5.5 cm) (5). The benefit of TEVAR cannot be definitively established due to the lack of randomized controlled trials (8-12). However, the endovascular technique is less complex, with good results in terms of reduced early mortality and complications, whereas conventional repair usually requires a more complex intraoperative and postoperative hospital structure. The advent of endovascular treatment options has resulted in a shift in the trends for the management of TAD (particularly descending thoracic aneurysms or dissections) since the early the 2000’s, and, nowadays, most groups are dedicated to endovascular repair procedures. Thus, TEVAR is a feasible and widespread technique that is fully covered by the Brazilian public health system.

Although differences in mortality rates of TAD are likely to exist between nations, current knowledge is limited to a few isolated studies from individual countries based on the results of specialized centers. In a study by Sidloff et al., which aimed to examine trends in mortality from thoracic aneurysms in several countries, the age-standardized mortality of TAD ranged from 0.5% in Japan to 9.7% in the Netherlands (13). Although mortality rates were evidently declining in some countries, the same tendency was not observed globally (13).

Data from high-volume centers indicate an in-hospital mortality of 7.8% for conventional thoracic aortic aneurysm repair (14) and approximately 2% for TEVAR (15). Patients subjected to TEVAR in an elective scenario have been shown to have better outcomes than those who present with rupture or ischemic complications (16).

There are 5,570 cities in Brazil, the largest of which is São Paulo, ranking 2^nd^ in the country's human development index (0.783) (17). In 2016, the population in São Paulo was estimated to surpass 12 million inhabitants (18), of which 5 million depended solely on the public health system (19).

To the best of our knowledge, there have been no studies published in Brazil that have focused on the evaluation of the outcome of endovascularly treated TAD in a large urban center over a long period of time and including several variables. This information is relevant for the understanding of the real-life scenario, as opposed to being restricted to those published by highly specialized centers.

In this study, we aimed to evaluate the endovascular management of isolated TAD (excluding traumas and congenital diseases) in 1,344 patients treated in the public health system in São Paulo between 2008 and 2019. We assessed the procedure type, frequency (elective or urgent), mortality, length of hospital stay, intensive care stay, and procedure costs.

## METHODS

This study was approved by the Institutional Ethics Committee under protocol 3067-17.

Public data concerning endovascular surgery procedures for isolated TAD (ITAD) repair performed from 2008 to 2019 were extracted from the TabNet platform of the Municipal Health Secretary of São Paulo. TabNet belongs to the Department of Informatics of the Unified Health System (DATASUS) (20), and renders open data on procedures performed by the Brazilian public health system. All data displayed through the DATASUS system are properly de-identified. The search was performed on May 24, 2020, and excluded thoracoabdominal procedures.

A Unified Procedure Table Management System (SIGTAP) systemizes sanitary institutions linked to the Unified Health System *Sistema Único de Saúde* (SUS) that are qualified to perform procedures of specific medical specialty, and ultimately represents an invaluable tool for the financial decision-making process. SIGTAP accreditation is a prerequisite for receiving government remittances for the procedures performed. For the purpose of this analysis, only SIGTAP accredited hospitals of “high complexity” in vascular surgery were considered.

The DATASUS platform allows 22 possible selections for rows, 16 for columns, and 8 for content, providing 2,816 possible formatting combinations, separated into monthly periods. In this study, the variables selected were sex, age group, city of residence, number of surgeries performed by establishment, in-hospital mortality, length of hospital stay, mean length of intensive care unit (ICU) stay, and reimbursement values paid by the SUS over the study period.

All endovascular procedures that were performed for the treatment of thoracic aortic aneurysms were analyzed. Procedures were identified using the SUS System of Procedures and Medicines coding for endovascular aneurysm repair/thoracic aortic dissection with a straight/conical stent graft (code: 04.06.04.017-6). Unfortunately, the system coding for both the diagnosis (ICD classification) and treatment procedure (procedural coding) are superimposed for aneurysms and dissections, rendering the separation of cases infeasible.

Procedures classified as “Q25” by the ICD were rejected in order to avoid the inclusion of procedures for correction of congenital malformations of the great arteries. Hospitalizations due to trauma (ICDs starting in ‘S’) were also excluded.

The selected cases were divided into two groups according to the hospitalization regimen reported: the elective group, consisting of patients who underwent surgery on an elective basis, and the urgent group, consisting of patients who underwent urgent hospital admissions.

All information was obtained from public access websites through content access programs using web scraping codes. Manual data collection processes, although technically feasible, would have been too time-consuming. Automatic navigation codes with programming assistance were used to facilitate and expedite data collection. These codes were programmed in Python language (v. 2.7.13, Beaverton, Oregon, USA) using the Windows 10 Single Language operating system. The stages of data collection, platform field selection, and subsequent table adjustments were performed using the Selenium WebDriver packages (v. 3.1.8, Selenium HQ, various contributors around the world) and pandas (v. 2.7.13, Lambda Foundry, Inc. and PyData Development Team, New York, USA). The web scraping code has a 14-search phase main structure (Appendix), which is adaptable to the different filters available on the platform. The Mozilla Firefox browser (v. 59.0.2, Mountain, California, USA) and WebDriver GeckoDriver (v 0.18.0, Mozilla Corporation, Bournemouth, England) were used during data collection.

After data collection and cleaning, all data were organized and grouped in spreadsheets using Microsoft Office Excel 2016^®^ software (v. 16.0.4456.1003, Redmond, Washington, USA). The tables were formatted so that the procedure type groups were placed side by side, and contained, for each group, the following subdivisions: The number of procedures performed in each of the establishments, the total number of operated patients, the in-hospital mortality (absolute and percentage), the length of hospital stay, the average length of ICU stay, and the remittance values paid by the SUS. All values are shown in Reais (R$, Brazilian official currency), which underwent a 68.75% inflation between 2008 and 2019, in accordance with data from the “ĺndice de Preços para o Consumidor Amplo” (IPCA). The included hospitals were numbered in descending order according to the total number of procedures that they performed. In our study, we were able to use the available public data in a simpler and quicker fashion to analyze a larger number of procedures and years.

The following tests were used for statistical analysis: The chi-square test to investigate whether there was a change between the number of patients treated in an elective or emergency setting over the study period (trend test); the Mann-Whitney test to compare, between the elective and urgent groups, the ordinal or continuous variables not normally distributed, the mortality rates, the hospitalization length, and the remittance values paid. The average length of ICU stay, given as the mean, was compared between groups using a generalized estimation equation with normal distribution and identity link function, assuming first-order autoregressive correlation between years in the same hospitals. For all tests, the level of statistical significance was set at *p*=0.05.

## RESULTS

In total, 1,344 endovascular procedures were performed in the city of São Paulo to correct ITAD between 2008 and 2019. The majority of patients treated were male (n=888; 66.07%) and <65 years of age (n=751; 55.87%). Nearly half of the patients treated for ITAD in São Paulo migrated from a different city (n=616; 45.84%), and the provenance was unknown for 58 patients.

The number, distribution percentage, and type of procedures (elective or urgent) per year are shown in Table 1. Elective procedures were more frequent, and although the trend test points to a significant tendency for an inversion in the proportion of procedure type distribution throughout the study period (*p*<0.001), this was not observed, especially in the final years. Two-thirds of the total procedures were performed in elective scenarios and one-third were performed in urgent scenarios, as shown in Table 1.

The mortality per procedure type by hospital is presented in Table 2. There were 128 in-hospital deaths (9.52% general mortality rate), and the in-hospital mortality was significantly lower for elective procedures (7.29%) than for urgent procedures (14.31%) (*p*=0.029). The hospital with the highest number of surgeries (n=323) had the lowest in-hospital mortality (5.26%).

The length of hospital stay is shown in Table 3. Most procedures were associated with a length of hospital stay longer than 7 days (59.67%), and this was maintained even when procedure types were analyzed separately. The length of hospital stay did not differ significantly between the groups (*p*=0.098).

**Figure 1:**
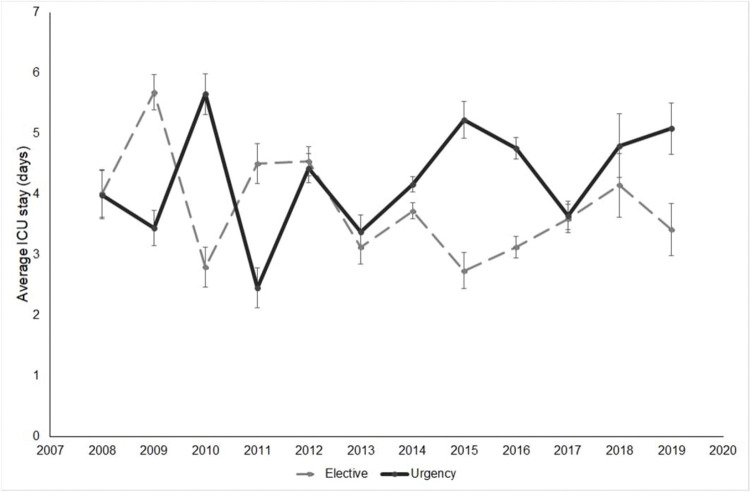
Average length of intensive care unit (ICU) stay in days by year in the elective and urgent treatment groups.

The average length of ICU stay (in days) by patient group per year is shown in Figure 1. Although the graph suggests an absence of tendency throughout the study period, with an apparent similar average between groups in the earliest years, the later years had an increased average length of ICU stay for the urgent group. Generalized estimation equation analysis showed that the mean ICU stay was similar between groups regardless of year (*p*=0.142). The distribution throughout the years was statistically similar for both groups (*p*=0.141), and there was no tendency for a shift between the average length of ICU stay of the procedure type groups during the study interval (*p*=0.735).

The reimbursement values paid by the public health system, by hospital establishment, in US dollars, are presented in Table 4. A total of R$ 24.766.008,61 was paid for all procedures. Considering the average value paid per procedure, the elective procedures cost an average of R$ 17.222,98, and urgent procedures cost an average of R$ 18.558,68; thus, urgent procedures were significantly more expensive (*p*=0.029).

## DISCUSSION

Approximately 75% of the Brazilian population is dependent on the SUS, a universal, equitable, and comprehensive tax-funded government system (21). The remaining 25% of the population makes use of the supplementary private health system, in which the costs are covered individually either by the user or their employer (19).

The city of São Paulo strays from this national pattern of distribution, mainly due to its financial status (22). In São Paulo, approximately 58.3% of the population (7 million people) have private health coverage, while 5 million depend solely on the public health system (19).

Our analyzed population consisted mainly of male patients (n=888; 66.07%), with 60.56% of the procedures (n=814) performed in individuals aged 65 years or older. The overall predominance of male patients aged over 65 years is in accordance with the expected population of TAD patients (23-25).

The studies of Howard et al. and Clouse et al. estimated a worldwide incidence between 5.6 and 10.4 cases per 100,000 patient-years for TAAs (26,27). Considering that approximately 5 million people are dependent on the public health system in São Paulo, it could be surmised that between 280 and 520 new cases of TAD will be diagnosed in São Paulo each year. Over the 12-year study period, the yearly average of operated patients was 112, which represents approximately 20% to 40% of the expected diagnostics. The proportion of diagnosed cases with indisputable surgical indication is unknown, and we are limited by the absence of the exact knowledge of the mortality rates of unoperated ITAD. However, it may be surmised that the intervention rate is lower than expected, especially because a vast proportion of patients who undergo surgery in São Paulo are, in fact, from different cities.

The delay in operating TAAs with definite indications for surgical correction may incur rupture. Several retrospective surveys indicate that rupture was the most common cause of death in patients with surgical-sized TAAs who were not operated on at the time of diagnosis, with death rates ranging from 42% to 74% (11). Thus, it is reasonable that, in a well-structured system, elective procedures should surpass urgent procedures in terms of volume. Our data show that in São Paulo, most TAD repair procedures in the past 10 years were conducted in an elective setting (68.3%). The trend test indicated a tendency towards an increase in the rate of urgent procedures over the study period (*p*<0.001), which points to an undesirable distribution. However, in the last evaluated year, the proportion is anomalously re-inverted, demonstrating a possible improvement in medical conduct. This observed distribution is in accordance with the international pattern, in which elective procedure rates range from 68.4% to 80% (23-25,28). The continued elevated quotient of procedures conducted in an urgent setting (31.7%) may indicate a need for greater investment in public care in São Paulo regarding TAD screening, in order to try to enhance detection in asymptomatic patients. Conversely, patients may be considered unfit for surgery on an elective basis and may only be operated on only when symptomatic. As we possess only database information, without granularity, we cannot truly rule out this possibility, which could be associated with even worse outcomes after urgent surgery.

The proportion of patients migrating from outside São Paulo for TAD treatment (45.84%) demonstrates an inclination for the centralization of health resources in a single urban center. Although the patients were not analyzed by procedure type based on provenance, we believe that the large number of patients whose treatment depended on being referred to and accepted by a larger center may have influenced the large proportion of cases that progressed to need urgent procedures.

The urgent group had a significantly greater mortality rate (14.31% *vs.* 7.29%, *p*=0.021). This finding is in accordance with a large study by Wang et al., who evaluated thoracic aortic aneurysm in the North American National Inpatient Sample, and found that elective status was protective against inpatient mortality (odds ratio [OR], 0.76; 95% confidence interval [CI], 0.58-0.99; *p*=0.042) (23).

The mortality rate of the elective group (7.29%) was similar to that described in a large series of cases by other authors (15), although some of these studies included more complex aneurysms in their samples. When analyzed individually, the institutions with the lowest mortality rates (5.26% and 6.07%) were those that performed the largest number of procedures (n=323 and n=313, respectively). This finding suggests that high-volume centers may, in fact, have a lower frequency of complications, as has been previously proposed for the treatment of open abdominal aortic aneurysm repair (29) and coronary artery bypass grafting (30,31). Conversely, the higher mortality rates observed for urgent (18.89%) procedures in our sample were observed in the analyzed hospital with the lowest considerable operatory volume.

The length of hospital stay has been previously determined to be a useful measure of health care efficiency, and in the current study, was similar for both groups (*p*=0.098) (32,33). In accordance with the findings of previous studies, most patients were hospitalized for 7 or more days (54.02%), although these authors found no difference between elective and emergent cohorts (24,25,34).

The length of ICU stay, as detailed in Figure 1, was 3.7 days on average for elective patients and 4.2 days on average for urgent patients, with no significant difference between groups (*p*=0.142). A retrospective analysis of the American College of Surgeons database published in 2019 found that emergency surgery was associated with longer ICU stays, as well as other factors such as aneurysm rupture or postoperative pneumonia (35). Conversely, in the study by Knowles et al. (24), the length of ICU stay was similar for patients who were operated in emergency and elective settings. One possible explanation for this similarity between groups is that patients who died precociously, in the early days of the ICU stay, may have lowered the average length of stay, likening the averages between the groups.

The reimbursement values paid for urgent procedures were significantly higher than those paid for elective cases (R$ 17.222,98 per elective case *vs.* R$ 18.558,68 per urgent case, *p*=0.029). However, this analysis is restricted by the fact that the public health system compensation table is often lower than the actual amount spent on the procedures, meaning that the true expenditure per procedure by the hospitals may in fact have surpassed the values documented by the public database.

Overall, our findings suggest that the institution of elective treatment for TAD patients is beneficial in terms of both mortality rates and procedure cost.

### Limitations

As is common with retrospective analyses, our study is limited by the loss of patient information. Furthermore, given that very few institutions in Brazil focus on the conventional repair of TAD, these procedures were so few that they bore no analytical importance and were therefore not included. The most significant limitation of our study is the fact that the database used only compiles data of hospitals accredited to SIGTAP. This certainly incurred loss of data of procedures performed in hospitals without accreditation, which were not listed in the DATASUS database, especially in emergency scenarios. Additionally, there may have been a fraction of trauma cases who were operated on not because of atherosclerotic degeneration of the aorta, in the context of healthier patients (probably younger), but as a result of multisystemic or high-energy trauma. Conversely, the results relating to elective procedures are unlikely to be overestimated, as non-accredited hospitals are not reimbursed for operations, and very few (if any) elective cases were operated in hospitals that were not reimbursed for the procedure.

Due to databank information anonymization, patient follow-up was not possible, and all mortality data discussed refers exclusively to in-hospital deaths. As a result, we were unable to contribute information with respect to long-term mortality rates. Another consequence of data anonymization is the inability to individualize cases with regard to the length of ICU and hospital stay. Because patients were grouped in the database by fixed intervals (‘< 1 day’, ‘1 day’, ‘2 days’, ‘3-7 days’, and ‘> 7 days), a more detailed representation through time medians was rendered impossible.

Lastly, we were unable to provide input with regard to the need for reinterventions, which are relatively more frequent in the context of endovascular procedures. This secondary limitation is also attributable to the anonymization necessary for data availability from public and governmental sources.

These limitations notwithstanding, this is a comprehensive analysis of the public health systems management of TAD in the largest urban center in Brazil. Moreover, this study covers a 10-year period and includes over 1300 procedures; thus, we consider that this is a representative assessment of a real-world sample of TAD patients. The results of this study provide useful information on a public system and guidance for the allocation of health funds.

## CONCLUSION

In São Paulo, TAD interventions required a governmental investment of over R$ 24.766.008,61 over a 10-year period. Elective procedures were performed almost twice as frequently as their urgent counterparts, while urgency procedures were more expensive and associated with a longer time of hospitalization. This pioneering study design permits initial examination of the profile of TAD treatment in the city of São Paulo, and may provide evidence to guide monetary resource allocation by the health system.

## AUTHOR CONTRIBUTIONS

Portugal MFC was responsible for the manuscript composition, critical revision, manuscript approval and study accountability. Teivelis MP and Wolosker N were responsible for the manuscript conception, composition, critical revision, approval and study accountability. Silva MFA, Stabellini N, Amaro-Junior E and Szlejf C were responsible manuscript conception and approval, data collection, and study accountability. Fioranelli A was responsible for the manuscript conception, critical revision, approval and study accountability.

## Figures and Tables

**Table 1:** Absolute and relative frequency of endovascular procedures for TAA correction according to the degree of urgency from 2008 to 2019.

	Elective		Urgent			
	n	%	n	%	Total	*p* *
2008	86	68.25	40	31.75	126	<0.001
2009	110	74.82	37	25.18	147	
2010	141	73.43	51	26.57	192	
2011	87	45.31	33	54.69	120	
2012	81	72.97	30	27.03	111	
2013	94	68.61	43	31.39	137	
2014	77	71.29	31	28.71	108	
2015	65	60.18	43	39.82	108	
2016	49	56.32	38	43.68	87	
2017	55	59.13	38	40.87	93	
2018	37	64.91	20	35.09	57	
2019	36	62.06	22	37.94	58	
Total	918	68.3	426	31.7	1344	

* Trend chi-square test

**Table 2:** Absolute and relative frequencies of procedures (%) and mortality (%) by hospital unit.

Hospital	Total no. of procedures			Endovascular procedures					
		Total mortality		Elective			Urgent		
		n	%	Procedures	Mortality (n)	%	Procedures	Mortality (n)	%
1	323	17	5.26	275	10	3.64	48	7	14.58
2	313	19	6.07	306	18	5.88	7	1	14.29
3	225	34	15.11	225	34	15.11	0	-	-
4	172	16	9.30	73	5	6.85	99	11	11.11
5	113	8	7.08	18	0	0.00	95	8	8.42
6	105	17	16.19	15	0	0.00	90	17	18.89
7	91	16	17.58	6	0	0.00	85	16	18.82
8	1	0	-	0	-	-	1	0	0.00
9	1	1	100.00	0	-	-	1	1	100.00
Total	1344	128	9.52%	918	67	7.29%	426	61	14.31
p*					*0.029*				

* Mortality in the urgent group was significantly higher than that in the elective group (*p*=0.029), Mann–Whitney test.

**Table 3:** Absolute and relative frequency of endovascular procedures for TAA correction according to the degree of urgency from 2008 to 2019bsolute and relative frequencies of procedures according to the number of days (time) of hospitalization.

Length of stay	<1 day		1 day		2 days		3–7 days		>7 days		
	n	%	n	%	n	%	n	%	n	%	*p* *
Elective	11	47.83	27	60.00	121	92.37	220	64.14	539	67.207	0.098
Urgent	12	52.17	18	40.00	10	22.22	123	35.86	263	32.793	
Total	23	1.71	45	3.35	131	9.75	343	25.52	802	59.67	1344

* The number of days of hospitalization in the urgent group was significantly higher than in the elective group (*p*=0.098).

**Table 4:** Values passed by the SUS in Brazilian Reais per hospital establishment.

Hospital	Total amount	Amount paid per procedure type		Amount paid per procedure	
		Elective	Urgent	Elective	Urgent
1	5,553,057.12	4,670,970.19	882,086.93	16,985.35	18,376.81
2	5,669,685.38	5,525,436.78	144,248.60	18,056.98	20,606.94
3	4,256,748.39	4,256,748.39		18,918.88	
4	3,290,079.69	1,272,002.62	2,018,077.07	17,424.69	20,384.62
5	2,052,019.77	317,970.07	1,734,049.70	17,665.00	18,253.15
6	2,017,861.75	221,495.74	1,796,366.01	14,766.38	19,959.62
7	1,896,801.62	100,461.49	1,796,340.13	16,743.58	21,133.41
8	2,775.81		2,775.81		2,775.81
9	26,979.08		26,979.08		26,979.08
Total	24,766,008.61	16,365,085.28	8,400,923.33	17,222.98	18,558.68

*Cost per patient for urgent procedures was significantly higher than that for elective surgery (*p*=0.029); Mann–Whitney test.

## APPENDIX

Description of the algorithm developed in the applications:

1. Enter the procedures of interest in the table of the search setting

2. Define the items to display in table rows and columns

3. Define the data content of each box (total value of AIH’s paid, total amount, ICU nights, others)

4. Select the period of interest

5. Select the fields of interest in the “AVAILABLE SELECTIONS” session from TabNet

6. Enter the procedure in the field “Procedure Performed”

7. Submit data request

8. Wait until the full page loads

9. Request to download the table generated a CSV (Comma-separated values) format file

10. Wait until the full file downloads

11. Rename the file to include the procedure name

12. Remove note and subtitle information

13. Rearrange the tables for use in Excel or other software for the analysis

14. Return to the initial selection form
